# Characterizing Treatment Delays in Patients With HPV‐Negative Oropharyngeal Cancer

**DOI:** 10.1002/cam4.71748

**Published:** 2026-03-24

**Authors:** Alena Pauley, Solene Jeanine Fereira, Tammy Binh Pham, Vivian Vo, Hayden Guidry, Celia Ramsey, Theresa Guo

**Affiliations:** ^1^ University of California School of Medicine San Diego La Jolla California USA; ^2^ Rocky Vista University College of Osteopathic Medicine Ivins Utah USA; ^3^ Hanna and Mark Gleiberman Head and Neck Cancer Center, Moores Cancer Center La Jolla California USA; ^4^ Department of Otolaryngology‐Head & Neck Surgery University of California, San Diego La Jolla California USA; ^5^ University of California Davis School of Medicinse Sacramento California USA

## Abstract

**Background:**

Although the impact of increased time to treatment initiation (TTI) on outcomes for patients with oropharyngeal squamous cell carcinoma (OPSCC) has been well‐studied, a deeper understanding of the mechanisms underlying delay in patients with human papillomavirus (HPV)‐negative OPSCC is lacking in the current literature.

**Objective:**

To assess differences in sociodemographic factors and treatment timelines between patients with HPV‐negative OPSCC with shorter versus. longer TTI.

**Methods:**

Patients treated for HPV‐negative OPSCC at a single academic institution between 2013 and 2023 were retrospectively identified via chart review and dichotomized by the cohort median TTI (53.5 days; defined as the time from biopsy to first treatment initiation). Clinical timelines between delayed and nondelayed patients were compared using descriptive statistics and Mann–Whitney *U* testing. Independent predictors of delayed TTI (> 53.5 days) were evaluated using multivariate logistic regression modeling, with adjusted odds ratios (aORs) and 95% confidence intervals reported.

**Results:**

Seventy‐six patients were identified. On multivariable analysis, male sex (aOR 3.28; 95% CI 1.02–10.49), unmarried status (aOR 5.96; 95% CI 1.36–26.07), primary chemoradiation versus surgery (aOR 0.25; 95% CI 0.07–0.85), and biopsy available before arrival (aOR 4.08; 95% CI 1.32–17.36) were independently and significantly (*p*< 0.05) associated with delayed treatment initiation. Treatment timeline analysis revealed that both the interval from biopsy to referral and the interval from PET scan to treatment initiation differed significantly between delayed and nondelayed patients (*p*< 0.05).

**Conclusion:**

Primary nonsurgical treatment and lack of social support were found to be independently associated with treatment delay in patients with HPV‐negative OPSCC. These findings highlight opportunities for improving the care of HPV‐negative OPSCC at the specialty level.

## 1 Introduction

Delays in receiving cancer care have been linked to worse health outcomes [1, 2, 3, 4, 5], with a lag of just 4 weeks in surgical, radiologic, or systemic treatment associated with increased mortality rates [6]. For oropharyngeal squamous cell carcinoma (OPSCC), such delays are common due to often subtle or nonspecific early symptoms and the complex, multidisciplinary coordination required from primary care providers, surgical, radiation, and medical oncology specialists, as well as the patients themselves [7]. As a result, delayed diagnosis and treatment frequently occur even at advanced stages and are associated with worse outcomes [8, 9, 10].

Despite improvements in diagnostic and therapeutic approaches, OPSCC remains a significant public health burden with more than 60,000 new diagnoses and approximately 15,000 associated deaths annually, in addition to the long‐term functional disabilities experienced among survivors [11, 12, 13]. OPSCC is stratified into human papillomavirus (HPV)‐positive disease versus HPV‐negative disease, as these tumors have clinically distinct pathophysiology, natural history, treatment paradigms, and outcomes. While HPV‐related disease is responsible for the increasing incidence of oropharyngeal disease, HPV‐negative disease is associated with a worse prognosis, including higher rates of recurrence and mortality [14]. HPV‐positive OPSCC has a 5‐year survival rate of approximately 75% as compared to HPV‐negative OPSCC, which has a 5‐year survival rate closer to 50%, nearly double the rate of recurrence, and more frequent delays in treatment [15, 16, 17]. Given this substantially poorer prognosis at baseline, timely access to care is especially critical for patients with HPV‐negative OPSCC to improve survival and reduce disease burden.

Many sociodemographic factors have been implicated in delays in OPSCC treatment initiation, underscoring the complexity of this issue. Older age, female sex, limited medical literacy, lack of insurance, and racial and ethnic underrepresentation have all been associated with prolonged time to treatment initiation (TTI) [18, 19, 20]. These disparities in social determinants of health highlight the systemic barriers that many patients face in accessing timely care.

In addition to sociodemographic factors, several clinical characteristics and treatment modalities have been linked to delayed care, particularly among patients with HPV‐negative OPSCC. Primary treatment with radiation therapy or other nonsurgical approaches is associated with longer TTI and decreased overall survival, even after adjusting for age, comorbidities, and tumor stage [16, 21]. Social factors such as limited support networks and increased social vulnerability have also been shown to delay postoperative radiation therapy [22, 23]. Conversely, surgery as the initial treatment is associated with more timely care, and surgical intervention within 67 days of diagnosis has been linked to significantly lower mortality risk [10].

While there is considerable literature on the impact of treatment delays on health outcomes, the specific treatment timelines—from initial biopsy to first treatment initiation—have not been well‐defined for HPV‐negative OPSCC patients. The substantial differences in clinical presentation, treatment response, and survival between HPV‐positive and HPV‐negative OPSCC necessitate separate evaluation of HPV‐negative disease, even within a small, single‐institution cohort. This distinction is particularly important given its worse prognosis and the possibility that delays in care may be more clinically consequential in this high‐risk population [24]. Clarifying these care timelines and the factors that contribute to delay is essential for identifying actionable targets for system‐level improvement. In a retrospective analysis of patients with HPV‐negative OPSCC at a single institution academic hospital, this study seeks to define treatment timelines and identify key factors contributing to delays that could be targeted to improve care delivery.

## 2 Methods

### 2.1 Patient Cohort

This was a retrospective analysis of patients with HPV‐negative oropharyngeal cancer who received treatment at a National Cancer Institute (NCI)‐designated cancer center within an academic hospital system between 2013 and 2023. Electronic medical records of patients treated at Moores Cancer Center (MCC) at the University of California (UC) San Diego Health System were screened for study inclusion. All patients with biopsy‐proven HPV‐negative OPSCC who received treatment at MCC between 2013 and 2023 were included in this study, yielding 76 subjects. Patients were included irrespective of whether they received treatment for primary or recurrent cancer, and p16 immunochemistry was used to define HPV‐associated tumor status. Research work was conducted with approval from the University of California San Diego Institutional Review Board (Protocol # 200068) prior to commencing this study. A waiver for informed consent was granted through the IRB.

### 2.2 Outcome Measures

Demographic, social, and treatment‐related data were collected from the medical records of all 76 included subjects. Demographic factors included age, sex, insurance status, alcohol, and smoking history, and social factors included an individual's marital status, relationship to their emergency contacts, and whether they lived alone at the time of their first MCC appointment. Clinical data included American Joint Committee on Cancer (AJCC) 8th edition staging, primary tumor site, dates of pertinent workup timepoints (e.g., biopsy, referral, imaging), dates of first appointments with a head‐and‐neck surgeon, medical oncologist, and radiation oncologist, date of primary treatment initiation, and primary treatment modality (surgery versus chemoradiation).

All patients were included in the analysis, even when their care did not follow a uniform clinical sequence. Rather than assuming a predefined pathway, timelines were constructed using documented milestone dates within the medical record. Time intervals between key events (e.g., referral to biopsy, biopsy to primary treatment) were calculated for each patient regardless of event order. These intervals were then analyzed in aggregate. Multidisciplinary tumor board meetings were not selected as an anchor within the treatment timelines as their timing and documentation were not reliably captured in this patient cohort's medical record. Referral was defined as the first documented referral to a head and neck cancer specialist within the treating institution. This was included as a treatment milestone regardless of whether a diagnostic biopsy had yet been performed. If multiple biopsies were required, the biopsy that established a diagnosis of malignancy was used in the analysis.

The median length of time from biopsy to primary treatment initiation in this cohort was 53.5 days, and this was used as the cutoff to delineate patients as having either delayed (≥ 53 days) or nondelayed (< 53 days) treatment. The median length of time was chosen over the mean to prevent skewness of the overall dataset, given the relatively small study population.

### 2.3 Statistical Analysis

Statistical analyses were performed in *R* version 4.4.1 [25]. Comparisons of demographic and social factors across patients with delayed versus nondelayed treatment timelines were analyzed using chi‐squared analyses. Comparisons of clinical treatment timelines across groups were analyzed using the Mann–Whitney *U* test. All testing was performed with an alpha level of 0.05. Treatment timelines between groups were illustrated graphically using LucidChart. Median time intervals between clinical time points were used for display and statistical analyses, given the decreased susceptibility to skewness and outlying variables. Missing data were excluded from all analyses.

To evaluate independent predictors of treatment delay, multivariable logistic regression modeling was performed with treatment delay (≥ 53 days) as the outcome variable. Covariates included sex, marital status, living alone status, surgery as the first treatment modality, and biopsy availability prior to arrival. Variables were selected a priori based on clinical relevance. Adjusted odds ratios (ORs) with 95% confidence intervals (CIs) were calculated. Statistical significance was defined as *p* < 0.05.

## 3 Results

A total of 76 patients met inclusion criteria. Most patients were male (68.4%), white (77.6%), and received treatment for an advanced‐stage (AJCC‐staging IV or higher) tumor (57.9%) (Table 1). The primary tumor site was limited to the base of the tongue in 44.7% of patients, the tonsil in 32.9% of patients, and the palate in 7.9% of patients, with the remaining 14.5% of tumors originating from overlapping sites or unknown primary (Table 1).

**TABLE 1:** Characteristics of Patients With and Without Treatment Delay.

	All patients, *N* = 76	No delay, (< 53 days to care), *N* = 38^a^	Delay, (≥ 53 days to care), *N* = 38^a^	Chi‐squared statistic	*p*
Age at Diagnosis	63.21	65.74	62.44	0	1
Sex				4.828	0.028
Male	52 (68.4%)	21 (55.3%)	30 (79.0%)		
Female	24 (31.6%)	17 (44.7%)	8 (21.1%)		
Race, *unknown: 1*				0.291	0.589
White	59 (77.6%)	30 (79.0%)	28 (73.7%)		
Non‐White	16 (21.1%)	8 (21.1%)	10 (26.3%)		
AJCC Stage, 8th edition, *unknown: 5*				0.001	0.973
Early (Stage I, II, and III)	27 (35.5%)	14 (36.8%)	13 (34.2%)		
Late (Stage IV)	44 (57.9%)	23 (60.5%)	21 (55.3%)		
T Category, *unknown: 3*				3.638	0.457
T0	2 (2.6%)	1 (2.6%)	1 (2.5%)		
T1	15 (19.7%)	8 (21.1%)	7 (18.4%)		
T2	20 (26.3%)	12 (31.6%)	8 (21.1%)		
T3	18 (23.7%)	6 (15.8%)	12 (31.6%)		
T4	18 (23.7%)	11 (29.0%)	7 (18.4%)		
N Category				3.279	0.194
N0	25 (32.9%)	15 (39.5%)	10 (26.3%)		
N1	16 (21.1%)	5 (13.2%)	11 (29.0%)		
N2	35 (46.1%)	18 (47.4%)	17 (44.7%)		
M Category				1.416	0.234
M0	69 (90.8%)	36 (94.7%)	33 (86.8%)		
M1	7 (9.2%)	2 (5.3%)	5 (13.2%)		
Packs*Year, *unknown: 7*				0.048	0.826
0–10 pack*year hx	28 (40.6%)	16 (43.2%)	13 (40.6%)		
> 10 pack*year hx	41 (59.4%)	21 (56.8%)	19 (59.4%)		
Insurance Status at Diagnosis				2.487	0.115
Private	23 (30.3%)	13 (34.2%)	11 (29.0%)		
Medicaid/MediCal	51 (67.1%)	23 (60.5%)	27 (71.1%)		
Uninsured	2 (2.6%)	2 (0.5%)	0 (0.0%)		
Psychological Comorbidities, *unknown: 4*				0.234	0.629
Yes	27 (35.5%)	13 (36.1%)	15 (41.7%)		
No	45 (59.2%)	23 (63.9%)	21 (58.3%)		
Alcohol Exposure, *unknown: 7*				0.118	0.731
Heavy	28 (38.9%)	14 (41.2%)	13 (37.1%)		
None or Occasional	41 (56.9%)	20 (58.8%)	22 (62.9%)		
Marital Status				6.373	0.012
Married/Partnered	36 (47.4%)	24 (63.2%)	13 (34.2%)		
Single/Widowed/ Divorced	40 (52.6%)	14 (36.8%)	25 (65.8%)		
Lives Alone at First Appointment, *unknown: 1*				4.321	0.038
Yes	27 (36.0%)	9 (24.3%)	18 (47.4%)		
No	48 (64.0%)	28 (75.7%)	20 (52.6%)		
Primary Tumor Site				1.424	0.840
Tonsil	25 (32.9%)	13 (34.2%)	12 (31.6%)		
Base of Tongue	34 (44.7%)	15 (39.5%)	18 (47.4%)		
Palate	6 (7.9%)	4 (10.5%)	2 (5.3%)		
Overlapping	8 (10.5%)	5 (13.2%)	4 (10.5%)		
Unknown Primary	3 (4.0%)	1 (2.6%)	2 (5.3%)		
Biopsy Performed Before Arriving at Cancer Center				4.343	0.037
Yes	55 (72.4%)	24 (63.2%)	32 (84.2%)		
No	21 (27.6%)	14 (36.8%)	6 (15.8%)		
Recurrent Disease				0.157	0.692
Yes	7 (9.2%)	3 (7.9%)	4 (10.5%)		
No	69 (90.8%)	35 (92.1%)	34 (89.5%)		
First Treatment Received				4.828	0.028
Surgical	24 (31.6%)	17 (44.7%)	8 (21.1%)		
Nonsurgical	52 (68.4%)	21 (55.3%)	30 (79.0%)		
Participated in Clinical Trial				1.024	0.312
Yes	23 (60.5%)	13 (34.2%)	9 (23.7%)		
No	53 (76.7%)	25 (65.8%)	29 (76.3%)		

^a^ n (%).

### 3.1 Characteristics of Patients With Delayed Versus Nondelayed Care

Demographic and social factors were compared between patients receiving delayed and nondelayed care. Male gender, being unmarried, living alone at their first appointment, receiving their initial biopsy outside of the cancer center, and not receiving surgery as their first treatment were all factors found to be significantly associated with treatment delay on chi‐squared analysis (*p* < 0.05; Table 1).

In multivariate modeling, the majority of associations observed in univariate analysis persisted, with four of these five variables remaining independently significant (*p* < 0.05; Table 2). Living alone at the first appointment was no longer significant after adjustment, likely due to collinearity with marital status. All other evaluated variable distributions, including race, smoking history, alcohol use history, insurance status, and primary tumor site, were similar between delayed and nondelayed patients. Tumor category (T category) and overall stage did not significantly differ between patients with and without delays to care. Participation in a clinical trial was not associated with delays in care.

**TABLE 2:** Multivariable Logistic Regression Modeling of Patients With and Without Treatment Delay.

	Adjusted OR	95% CI	*p*
Male sex	3.28	1.02–10.49	0.045
Single marital status	5.96	1.36–26.07	0.018
Lives alone	0.56	0.12–2.51	0.451
Surgery as first treatment	0.25	0.07–0.85	0.028
Biopsy available prior to arrival	4.80	1.32–17.39	0.017

### 3.2 Comparison of Treatment Timelines

**FIGURE 1:**
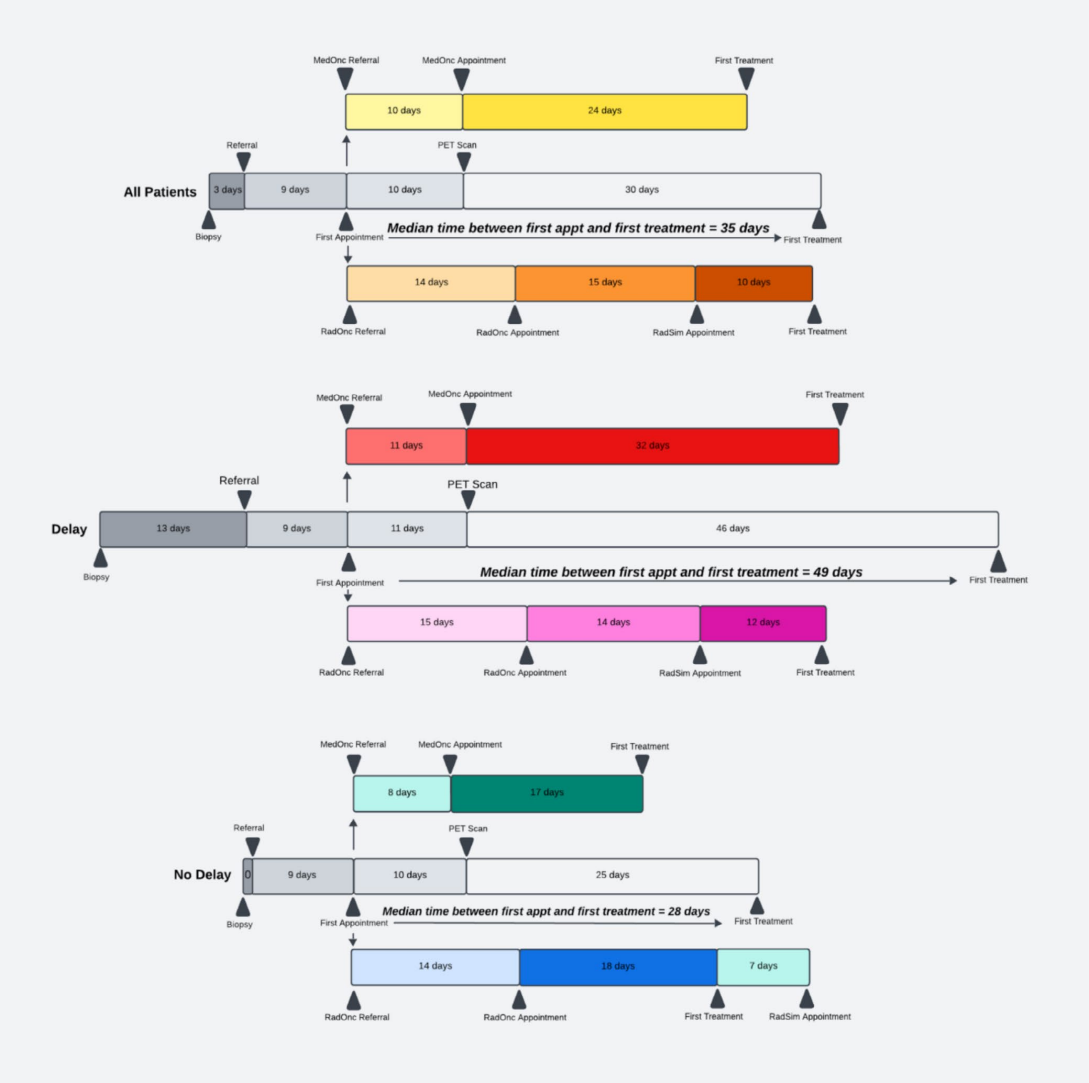
Treatment Timelines for Patients Receiving Delayed and Nondelayed Care.

The median amount of time between major clinical care milestones was 4 days from biopsy to referral, 9 days from referral to the first appointment, and 10 days from the first appointment to Positron Emission Tomography (PET) scan, respectively (Figure 1). The time from the PET scan to receiving the first treatment was the longest gap at 30 days.

These timelines were further stratified by patients with delayed vs. nondelayed treatment, as delineated by the 53‐day median length of TTI. When subdivided, the most variable times were from biopsy to referral, where the median was 0 days for nondelayed patients but 13 days for delayed patients, and the time from PET scan to treatment, where the median was 25 days for nondelayed patients and 46 days for delayed patients (Figure 1). There were little to no observed differences between groups in the time from referral to the first appointment and from the first appointment to their first PET scan.

The differences in time between biopsy to referral and first appointment to first treatment were found to be statistically significant (*p* < 0.05) between patients with and without treatment delay (Table 3), while this was not the case for all other time points.

**TABLE 3:** Difference in Median Timepoints in Patients With and Without Treatment Delay.

Median Days	All patients, *N* = 76	No delay, (< 53 days to care), *N* = 38^a^	Delay, (≥ 53 days to care), *N* = 38^a^	*Z*‐score	*p*
Timepoints					
Biopsy to Referral	3	0	13	3.384	< 0.001
Referral to 1st Appt	9	9	9	0.314	0.757
Referral to 1st Treatment	44	38	62	3.643	< 0.001
1st Appt to PET Scan	10	10	11	0.276	0.779
1st Appt to 1st MedOnc Appt	11	11	11	0.058	0.952
1st Appt to 1st Rad Appt	19	17	19	0.657	0.509
1st Appt to 1st Treatment	35	28	49	3.652	< 0.001
Biopsy to Treatment	53.5	42	76	7.500	< 0.001

^a^ Mann‐Whitney *U* test between nondelayed and delayed patients.

### 3.3 Surgical Versus Nonsurgical Care Pathways

**FIGURE 2:**
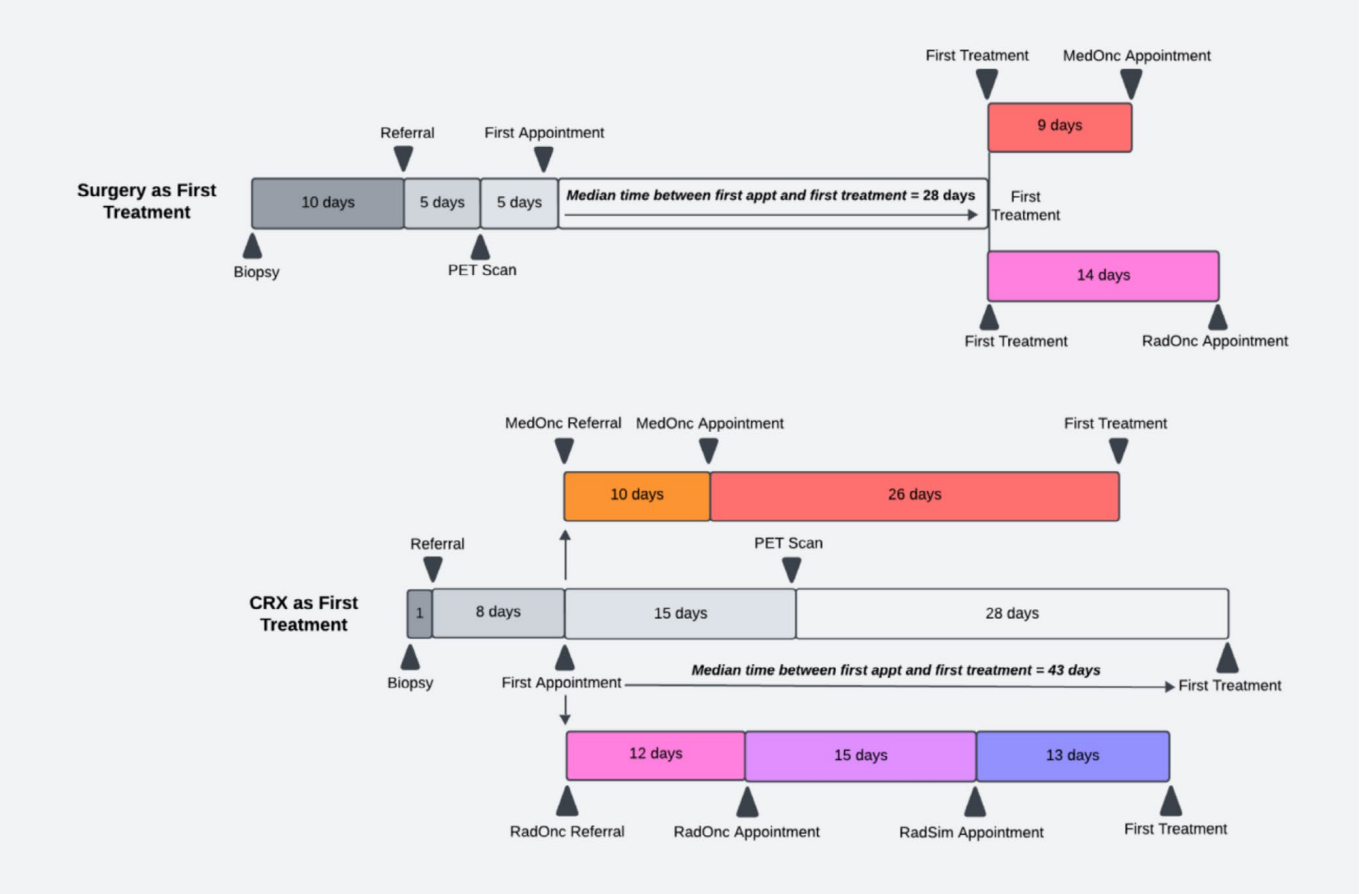
Treatment Timelines for Patients with Surgery versus CRT as First Treatment.

To better characterize differences in clinical care processes, we compared treatment timelines between the 24 patients who received surgery as their first treatment versus the remaining 52 patients who first underwent chemoradiation therapy (Figure 2). The median time from initial appointment to treatment initiation was shorter for the surgical group (28 days) than for those who received chemoradiation (43 days).

Among patients treated surgically, although a more prolonged interval was observed between biopsy and referral, subsequent workup, such as PET imaging, was completed earlier in the treatment course. Consultations with medical and radiation oncologists typically occurred after surgical intervention, rather than as part of the pretreatment evaluation. Finally, patients in the chemoradiation pathway had additional steps in their care timeline, such as the radiation simulation appointment, which introduced additional points of delay.

## 4 Discussion

This study provides a thorough comparison of patient characteristics and clinical timelines between patients who received delayed versus nondelayed treatment for HPV‐negative OPSCC at an NCI‐designated cancer center and identifies key factors associated with delayed care. Although smaller than prior population‐based studies, this focused, single‐institution cohort enables a granular reconstruction of care timelines that is often not possible in larger datasets and may otherwise be obscured. The information presented here is of critical use to guide and inform specific interventions to reduce TTI for patients with HPV‐negative OPSCC.

### 4.1 Social Vulnerability

We found that key sociodemographic factors associated with treatment delay in the final multivariate analysis included male sex and indicators of limited social support, such as being unmarried. While living alone was not found to be an independent risk factor for delay, this likely was a covariate with marital status, as 96% of patients living alone were also unmarried. This finding aligns with existing evidence linking social vulnerability to poor healthcare access and health outcomes, such as fewer cancer screenings, delayed initiation of needed therapies, and worsened survival rates [26, 27, 28, 29, 30, 31]. Social support is of special importance in our study population as patients with HPV‐negative OPSCC have been found to have heightened psychological distress and higher rates of anxiety and depression stemming from their diagnosis as compared to those with HPV‐positive tumors [32]. Social support may play an important role in navigating the complicated and demanding course of clinical treatment for OPSCC, as well as the significant toll this diagnosis and subsequent treatment can take on a patient's emotional and psychological well‐being. These observed associations suggest a potential need for greater support for these patients throughout treatment, and greater attention for those with less social support at baseline.

Interestingly, our finding that men experienced longer TTI contrasts with other national studies of HPV‐negative OPSCC, which have reported shorter TTI among male patients [33, 34]. This discrepancy may reflect the smaller sample size and regional focus of our study, which centers on a specific population in San Diego rather than a broad national cohort. Overall, this analysis helps to illustrate the important role that family members and loved ones play in patient care, and highlights that those who are male and unpartnered, and live alone, may be important populations to consider for patient navigation programs and in receiving support from the medical team to facilitate treatment and prevent delay [35, 36].

### 4.2 Treatment Pathways

Our multivariate‐adjusted results also demonstrated that patients receiving chemoradiation therapy as a first treatment experienced longer TTI compared to those treated with primary surgery, echoing previous studies and spotlighting an area for hospital‐level improvements in cancer care [37]. Primary surgical treatment has been consistently associated with shorter time to treatment [37, 38, 39] and improved 5‐year survival compared to chemoradiotherapy [38, 40]. The need to coordinate complex multimodal therapies can potentially contribute to these delays, and nonsurgical candidates may also have more advanced disease. In our dataset, among those who began with nonsurgical treatment, we observed a notable lag between referral to radiation oncology and the start of treatment. A closer review identified delays between patients' first radiation oncology appointment and simulation visit as a source of treatment initiation delays (Figure 2), which may partially account for the observed differences in surgical and chemoradiation TTI. These findings underscore the need for special attention to patients undergoing chemoradiation, who may be more susceptible to treatment delays given the greater complexity of their care pathway. This further presents an opportunity for hospital‐level process evaluation and future quality improvement efforts focused on streamlining and accelerating the chemoradiation treatment.

Lastly, the location of the biopsy was also independently associated with delayed treatment in this cohort. Patients who had their biopsy performed outside the cancer center experienced longer TTI, likely reflecting multifactorial delays during the transition between health centers. Some of this delay may be influenced by where the patients entered the cancer center's care; those referred before biopsy may appear to have shorter TTI because diagnostic processes occurred in‐house. Of note, these metrics do not capture earlier, upstream care delays, such as the time from symptom onset or initial healthcare contact to treatment, which are difficult to measure retrospectively but may nevertheless be influential determinants of disease progression. In addition, symptom onset, in both retrospective and prospective data collection, can be highly subjective, making the measurement of delay from symptom onset difficult. Often, initial healthcare contact is also difficult to ascertain, as these visits are with external providers. Therefore, although imperfect, we utilized the time of tissue diagnosis for our analysis.

Previous studies have similarly demonstrated that treatment timelines for oropharyngeal cancer vary based on patients' entry points into the healthcare system and the number of providers seen before diagnosis [7, 41, 42, 43]. Still, for patients starting their workup externally, opportunities exist to improve coordination, for example, through timely referrals, efficient transfer of diagnostic data, and stronger communication between institutions. Strengthening these transitions of care may help prevent unnecessary delays tied to care fragmentation.

### 4.3 TTI Delay and Survival Outcomes

Our findings that male sex, limited social support, and treatment pathway are associated with longer TTI should be interpreted in the context of prior literature demonstrating that shorter TTI is associated with improved patient outcomes [10, 44]. Across cancer types, treatment delays have been consistently associated with worse outcomes [1, 2, 3, 4, 5] and higher mortality rates [8, 9, 10]. In head and neck cancer specifically, a TTI threshold of 60 days has been identified as predictive of overall survival [29]. Targeting faster TTI is particularly critical for patients with HPV‐negative OPSCC, who not only experience poorer overall survival but for whom TTI has been shown to significantly influence prognosis, with the strongest effects observed in this subgroup [13, 45]. As such, developing and implementing robust interventions that can reduce treatment timelines from biopsy to treatment may inform broader improvements in patient care, regardless of sociodemographic factors. It is critical to foster shortened clinical timelines for the treatment of OPSCC when possible, but to be successful, these process improvement initiatives should be cognizant of factors underlying treatment delay.

### 4.4 Limitations, Strengths, and Future Directions

While this manuscript presents critical information on treatment timelines for patients with HPV‐negative OPSCC, several limitations must be considered. First, this is a smaller retrospective patient cohort from a single academic research institution, which limits generalizability. The narrow focus on HPV‐negative OPSCC patients presenting to one center increases the potential for selection bias and may not fully capture variations in care that exist across different healthcare systems. In addition, our analysis was unable to account for care delays prior to the first biopsy, which may independently influence these oncologic outcomes.

As such, while the timeline delays identified here are likely relevant to other settings, they may not universally reflect broader patient experiences or care pathways. However, the smaller sample size was well‐suited to this exploratory analysis, enabling a more detailed, nuanced understanding of care timelines in a high‐risk population often underrepresented in large‐scale datasets. Focusing on a single institution allowed for deeper insights into local care processes, laying the groundwork for targeted process improvement efforts and future multicenter research. Our nuanced institution‐level depiction of care timelines in HPV‐negative OPSCC highlights specific, potentially modifiable system factors that may inform future prospective and multicenter studies. Given the significance of the findings presented here, future studies should seek to validate these results across larger and more diverse populations to better inform systemic interventions aimed at reducing treatment delays and improving outcomes.

## 5 Conclusion

This analysis provides an in‐depth comparison of clinical timelines between patients receiving delayed versus nondelayed treatment for HPV‐negative OPSCC and highlights key factors associated with clinical delay. We found that longer treatment initiation times were associated with markers of limited social support and receipt of chemoradiation as first‐line therapy. These findings suggest that chemoradiation pathways may introduce delays in patient care and thus may need further interventional support to ensure efficient care. Our findings further suggest that patients with demonstrated poor social support may warrant closer clinical follow‐up to achieve the same outcomes as their peers. This analysis should be used to help inform future interventions aimed at improving care for HPV‐negative OPSCC.

### 5.1 IRB Approval

Research work was conducted with approval from the University of California San Diego Institutional Review Board (Protocol # 200068) prior to commencing this study. A waiver for informed consent was granted through the IRB.

## Author Contributions

**Alena Pauley:** data curation (equal), formal analysis (equal), investigation (equal), methodology (equal), visualization (lead), writing – original draft (lead), writing – review and editing (equal). **Solene Jeanine Fereira:** data curation (equal), formal analysis (equal), investigation (equal), methodology (equal), writing – review and editing (supporting). **Tammy Binh Pham:** formal analysis (equal), funding acquisition (equal), investigation (equal), methodology (equal), supervision (supporting), validation (lead), writing – review and editing (lead). **Vivian Vo:** data curation (supporting), writing – review and editing (supporting). **Hayden Guidry:** data curation (supporting), writing – review and editing (supporting). **Celia Ramsey:** data curation (supporting), writing – review and editing (supporting). **Theresa Guo:** conceptualization (lead), data curation (equal), formal analysis (equal), funding acquisition (lead), investigation (equal), methodology (equal), project administration (lead), resources (lead), supervision (lead), writing – review and editing (lead).

## Funding

This work was supported by Gleiberman Early Career Faculty Fellow.

## Conflicts of Interest

The authors declare no conflicts of interest.

## Data Availability

The data that support the findings of this study are derived from patient medical records at a single academic institution and are not publicly available due to institutional privacy policies and patient confidentiality concerns. De‐identified data may be made available from the corresponding author upon reasonable request and with appropriate institutional approvals.
